# Analysis of Flavonoids in *Dalbergia odorifera* by Ultra-Performance Liquid Chromatography with Tandem Mass Spectrometry

**DOI:** 10.3390/molecules25020389

**Published:** 2020-01-17

**Authors:** Xiangsheng Zhao, Shihui Zhang, Dan Liu, Meihua Yang, Jianhe Wei

**Affiliations:** 1 Hainan Provincial Key Laboratory of Resources Conservation and Development of Southern Medicine, Hainan Branch of the Institute of Medicinal Plant Development, Chinese Academy of Medical Sciences & Peking Union Medical College, Haikou 570311, China; xiangshengzhao@hotmail.com (X.Z.); 13006070475@163.com (S.Z.); yangmeihua15@hotmail.com (M.Y.); 2 School of Life Science and Engineering, Southwest University of Science and Technology, Mianyang 621010, China; 15280986611@163.com; 3 Institute of Medicinal Plant Development, Chinese Academy of Medical Sciences & Peking Union Medical College, Beijing 100193, China

**Keywords:** *Dalbergia odorifera*, flavonoid, assay, fragmentation behavior, UPLC-MS/MS

## Abstract

*Dalbergia odorifera*, a traditional Chinese medicine, has been used to treat cardio- and cerebrovascular diseases in China for thousands of years. Flavonoids are major active compounds in *D. odorifera*. In this paper, a rapid and sensitive ultra-high performance liquid chromatography-triple quadrupole mass spectrometry method was developed and validated for simultaneous determination of 17 flavonoids in *D. odorifera*. Quantification was performed by multiple reaction monitoring using electrospray ionization in negative ion mode. Under the optimum conditions, calibration curves for the 17 analytes displayed good linearity (*r*^2^ > 0.9980). The intra- and inter-day precisions (relative standard deviations) were lower than 5.0%. The limit of quantitation ranged from 0.256 to 18.840 ng/mL. The mean recovery range at three spiked concentrations was 94.18–101.97%. The validated approach was successfully applied to 18 samples of *D. odorifera*. Large variation was observed for the contents of the 17 analytes. Sativanone and 3′-*O*-methylviolanone were the dominant compounds. The fragmentation behaviors of six flavonoids were investigated using UPLC with quadrupole time-of-flight tandem mass spectrometry. In negative ion electrospray ionization mass spectrometry, all the flavonoids yielded prominent [M − H]^−^ ions. Fragments for losses of CH_3_, CO, and CO_2_ were observed in the mass spectra. Formononetin, liquiritigenin, isoliquiritigenin, sativanone, and alpinetin underwent retro-Diels–Alder fragmentations. The proposed method will be helpful for quality control of *D. odorifera*.

## 1. Introduction

*Dalbergia odorifera* T. Chen (Leguminosae) is a semi-deciduous perennial tree that is indigenous to Hainan Province, South China. It has been introduced to and cultivated in Guangdong, Guangxi, Fujian, and Yunnan provinces, China [1]. Heartwood of *D. odorifera* is an important traditional Chinese medicine called “*Jiangxiang*” that is widely used to resolve stasis, stanch bleeding, regulate *qi*, and relieve pain [2]. In Korea, this heartwood is also used for treatment of blood stagnation, ischemia, swelling, necrosis, and rheumatic pain [3]. In addition, *D. odorifera* is commonly used as a component of commercial drug mixtures for cardiovascular treatment, including Guan-Xin-Er-Hao decoction [4], Qi-Shen-Yi-Qi decoction [5], Xinning tablets [2], and Tongxinluo capsules [6]. Also known as fragrant rosewood (*Huanghuali* in Chinese), *D. odorifera* is a valuable wood product for manufacture of furniture, artifacts, and musical instruments [7]. Due to its high medicinal and commercial value, many researchers have studied *D. odorifera.* The strong market demands combined with the slow growth of *D. odorifera* have resulted in production of counterfeit items. To ensure the safety and efficiency of *D. odorifera* in clinical practice, quantitation of its functional components is critical.

Phytochemical investigations have demonstrated that flavonoids and volatile oils are the main medicinal components of *D. odorifera* [8]. Flavonoids are secondary polyphenolic metabolites occurring commonly in many medicinal plants. Due to their extensive pharmacological activities, flavonoids are considered as the active principle components in many herbs. Recent investigations have shown that flavonoids in *D. odorifera* possess various biological activities, such as anti-inflammatory [9,10], antioxidant [11], antitumor [12], antibacterial [13], and vasorelaxant activities [14]. Meanwhile, 3′-*O*-methylviolanone, sativanone, butein, liquiritigenin, butin, formononetin, etc. are the main compounds in *D. odorifera* [15,16,17]. Therefore, flavonoids could be considered as marker compounds to assess the quality of *D. odorifera*. However, no quantitative markers, except the content of its essential oils, have been selected for quality control of *D. odorifera* in the Chinese Pharmacopeia, which severely limits its clinical application and in-depth study. Among the analytical methods used for determination of flavonoids, the most widely used are based on high-performance liquid chromatography (HPLC) coupled with an ultraviolet (UV) or diode array detector [15,16,17]. The *D. odorifera* matrix is highly complex and the compounds of interest might be present in only minute quantities or accompanied by many other compounds with similar structures. In most cases, techniques like HPLC-UV will not be the optimum choice and can have long run times. A rapid, validated, and sensitive multi-component analytical method for quantification is required. 

Ultra-high performance liquid chromatography (UHPLC) combined with triple quadrupole mass spectrometry (QqQ-MS) is considered one of the most efficient techniques for quantitative analysis, and can provide specific, sensitive, and selective quantitative results in multiple reaction monitoring (MRM) mode [18]. Although numerous UHPLC-tandem mass spectrometry (MS/MS) methods have been applied to the determination of bioactivities components in traditional Chinese medicines [19,20], few studies have applied this method to quantitative analysis of flavonoids in *D. odorifera* [15,16,17,21]. Additionally, UHPLC-quadrupole time-of-flight (Q/TOF)-MS/MS has become increasingly important in compound identification because of its high selectivity, specificity, and accuracy [22].

**Figure 1:**
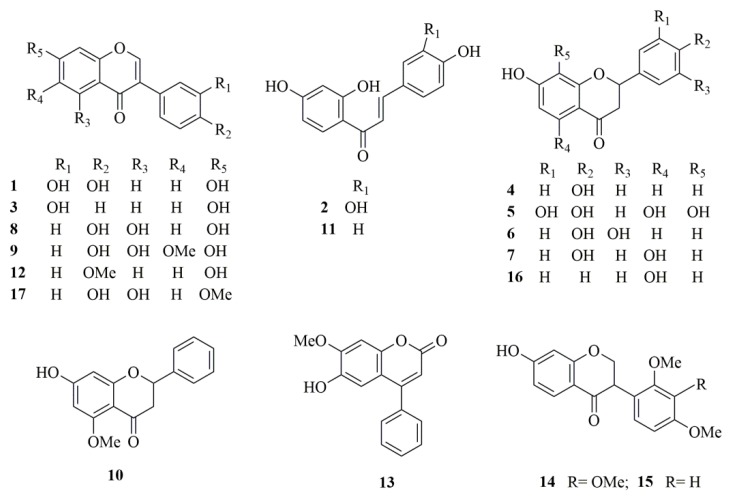
Chemical structures of the seventeen target compounds. 1, 3′-Hydroxydaidein; 2, Butein; 3, Daidzein; 4, Liquiritigenin; 5, Eriodictyol; 6, Butin; 7, Naringenin; 8, Genistein; 9, Tectorigenin; 10, Alpinetin; 11, Isoliquiritigenin; 12, Formononetin; 13, Dalbergin; 14, 3′-*O*-methylviolanone; 15, Sativanone; 16, Pinocembrin; 17, Prunetin. Analytes numbers in the test is the same as in this figure.

In the present study, a rapid and sensitive UHPLC-QqQ-MS method was established using MRM mode for the simultaneous quantitative analysis of 17 flavonoids (daidzein, dalbergin, 3′-hydroxydaidein, liquiritigenin, isoliquiritigenin, alpinetin, butein, naringenin, butin, prunetin, eriodictyol, tectorigenin, pinocembrin, formononetin, genistein, sativanone, and 3′-*O*-methylviolanone, Figure 1) in *D. odorifera* grown in different areas of China. The fragmentation behaviors of six different types of flavonoids were explored using UHPLC-Q/TOF-MS/MS in negative ion mode. This study is an example of comprehensive quality control and expands the knowledge of quantitative and qualitative analysis of multiple flavonoids in *D. odorifera*.

## 2. Results and Discussion

### 2.1. Method Development

To develop a sensitive and accurate quantitative method, the analytes and internal standard (IS) were separately infused into the instrument to optimize the mass conditions. MS spectra were investigated in both positive and negative modes. All analytes showed maximum sensitivity in negative ion mode. For optimization of the MRM conditions, the cone voltage and collision voltage were optimized to acquire the richest relative abundance of precursor and daughter ions. Two transitions were monitored for identification of each component, and the transition with the higher intensity was selected for quantification. The retention time and MS parameters for each analyte are presented in Table 1. 

**Figure 2:**
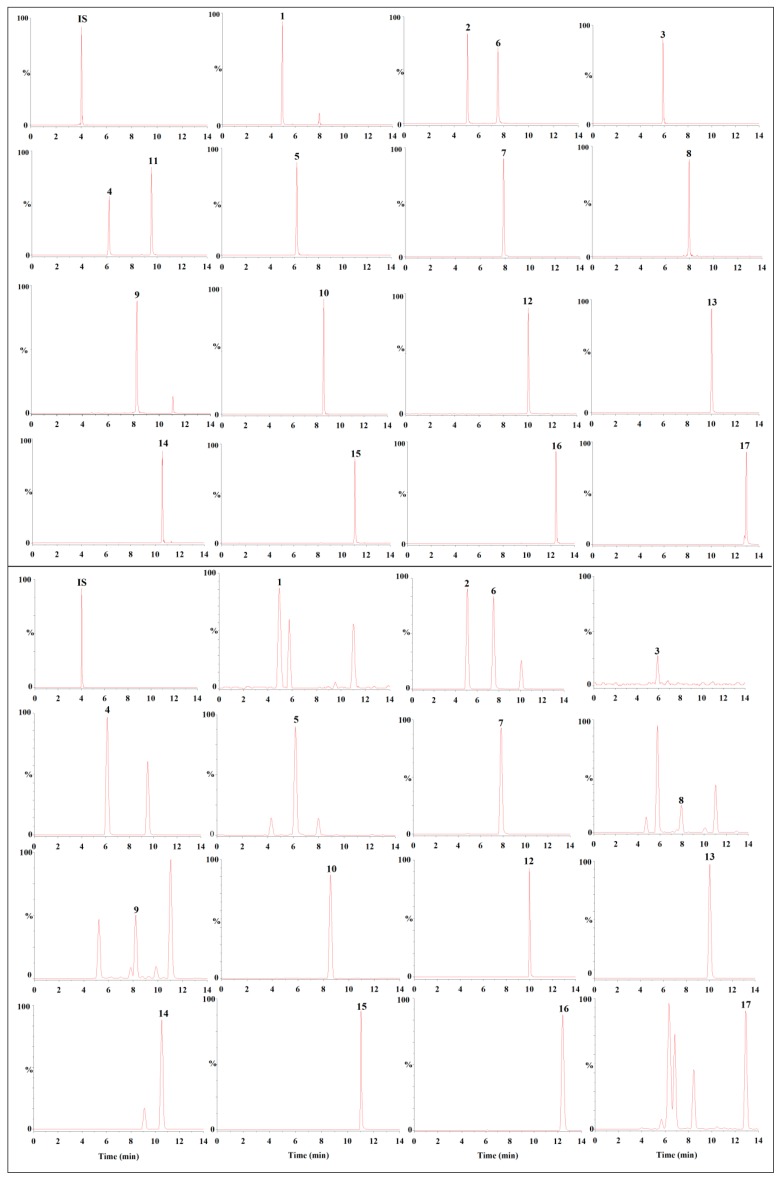
Ultra-high performance liquid chromatography (UHPLC)-MS/MS multiple reaction mode chromatograms of mixed standards (upper) and sample (lower, S9).

To optimize the chromatographic behavior, the UHPLC conditions were explored. First, a Waters Acquity BEH C18 column (100 mm × 2.1 mm, 1.7 mm) and Waters Acquity HSS T3 (100 mm × 2.1 mm, 1.8 μm) were examined. The Waters Acquity HSS T3 was chosen as it gave better separation and sharper peaks. Next, acetonitrile–water, methanol–water, and various additives (i.e., formic acid and acetic acid) were tested as potential mobile phases. Compared with the methanol–water system, the acetonitrile–water system gave better peak shapes and resolutions. For the modifiers, we found that acetic acid markedly inhibited the responses of the compounds. In addition, ionization of the flavonoids was inhibited if the concentration of formic acid was too high. Therefore, the concentration of formic acid was set at 0.05%. The effects of the column temperature, flow rate, and elution procedure were also investigated. Finally, acetonitrile containing 0.05% formic acid with a flow rate of 0.25 mL/min and the 40 °C column temperature were selected to achieve satisfactory separation in a short time (Figure 2). 

### 2.2. Optimization of the Extraction Conditions

**Figure 3:**
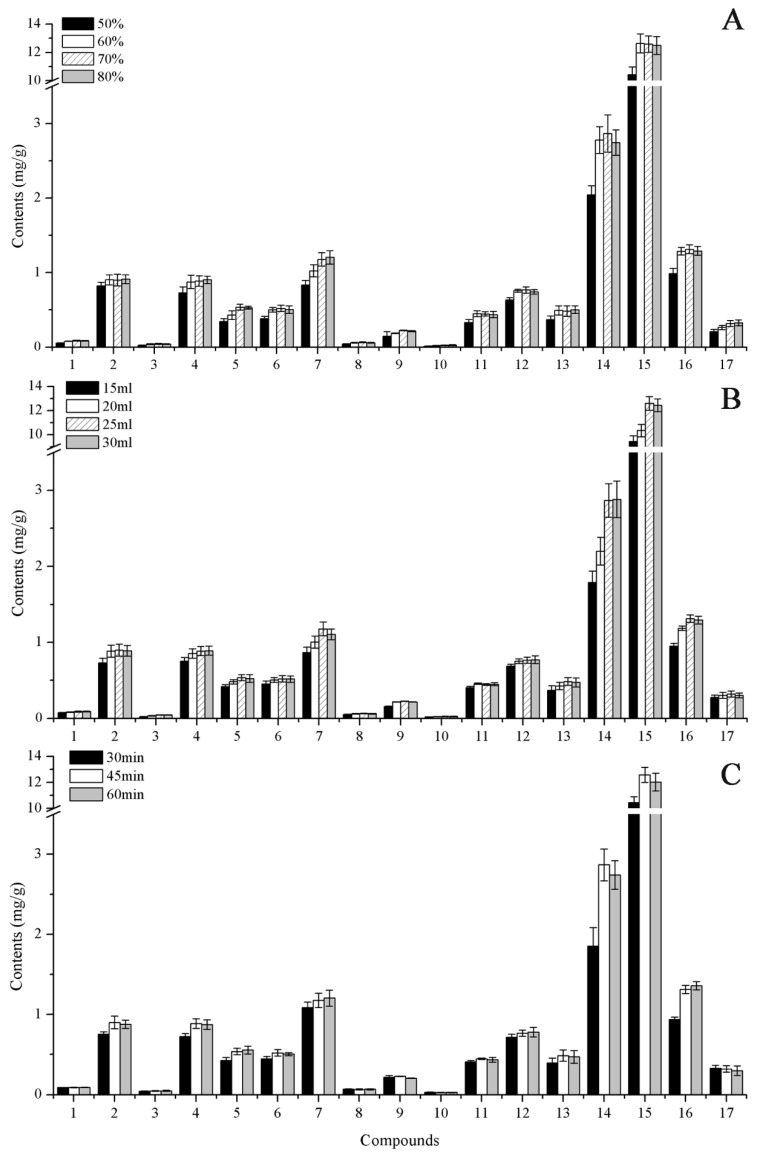
Effects of (**A**) solvent concentration, (**B**) solvent volume, and (**C**) extraction time on the extraction efficiency of target analytes in S9 sample.

Sample preparation methods are of key importance in the analysis of samples with complex matrices, and especially in the simultaneous analysis of multiple compounds. To develop an efficient and appropriate extraction method for the 17 target components for UHPLC-MS/MS analysis, reflux extraction and ultrasonic extraction were compared used sample S9 (0.2 g). The two extraction methods gave similar results but the ultrasonic extraction was more convenient (Figure S1). Thus, ultrasonic extraction was chosen for subsequent experiments. To optimize the extraction, the extraction solvent (50%, 60%, 70%, or 80% methanol, *v*/*v*), extraction volume (15, 20, 25, or 30 mL), and extraction time (30, 45, or 60 min) were investigated. When the methanol concentration was increased from 50–70%, the extraction efficiencies for the analytes increased (Figure 3A). However, when the methanol concentration was increased beyond 70%, the extraction efficiencies showed no large increases. Therefore, we chose 70% methanol as the extraction solvent. There were no obvious differences in the contents of analytes between extraction volumes of 25 and 30 mL (Figure 3B), and the contents of some compounds some compounds (e.g., eriodictyol, naringenin, 3′-*O*-methylviolanone, sativanone, and pinocembrin) were higher than 20 mL. The best extraction time for all components was 45 min (Figure 3C). Hence, the optimum conditions for extraction of *D. odorifera* were 0.2 g of dried sample, 25 mL of 70% methanol, and ultrasonic extraction for 45 min.

### 2.3. Method Validation 

The developed UHPLC-MS/MS method for quantitation of 17 flavonoids was validated to determine the specificity, linearity, limit of detection (LOD), limit of quantification (LOQ), intra- and inter-day precisions, stability, and accuracy according to International Conference on Harmonization (ICH) guidelines for validation of analytical procedures [23].

#### 2.3.1. Specificity

The representative MRM chromatograms of the mixed standard solution and real sample solution are shown in Figure 2. All of the target compounds could be distinguished using their retention times and precursor-to-product ion transitions. This indicates that the assay for *D. odorifera* is highly specific and selective. 

#### 2.3.2. Linear range, LOD, and LOQ

Linearity was evaluated using the coefficients of correlation (*r*^2^), which are listed along with the calibration curve equations, linear ranges, LOD, and LOQ in Table 2. Within the investigated concentration ranges, all compounds showed good linearity with *r*^2^ ranging from 0.9986 to 0.9999. The LOD and LOQ for each analyte were calculated using signal-to-noise ratios of three and ten, respectively. The LOD range was 0.085–6.080 ng/mL and the LOQ range was 0.256–18.840 ng/mL for the 17 target compounds, which showed the method had high sensitivity. 

#### 2.3.3. Precision, Repeatability, and Stability 

The intra- and inter-day variability were measured to assess the precision of the developed method using sample 9. The intra-day precision was evaluated by analyzing six replicates prepared from sample 9, and the inter-day precision was examined over three consecutive days with samples per day. The repeatability was determined by injection of six samples prepared following the same procedure (Section 2.4). The stability of the sample solution over 24 h at room temperature was also evaluated. For the precision, repeatability, and stability tests, the percent relative standard deviations were within 5.0% (Table 2). 

#### 2.3.4. Accuracy 

To further evaluate the accuracy of the proposed method, a recovery test was carried out by spiking three levels (80%, 100%, and 120% of the known amount) of mixture standard solution and corresponding IS standards to known amount samples. Next, the spiked samples were extracted and analyzed using the proposed method, and then, triplicate experiments were performed at each level. The recoveries were calculated using the following equation: Recovery (%) = (total amount detected − amount in original sample)/amount spiked × 100%. The recovery for each compound was in the range of 94.18–101.97% and the relative standard deviation was less than 6.0% (Table S1 in Supplementary Materials). The results implied that the developed UHPLC-MS/MS was precise, accurate, sensitive, and reliable enough for simultaneous quantitative analysis of the 17 target compounds in *D. odorifera*. 

### 2.4. Method Application

The validated method was applied to determine the 17 selected flavonoids in 18 samples of *D. odorifera*. Representative MRM chromatograms are shown in Figure 2 and the quantitative results are shown in Table 3. The contents of the 17 analytes varied in different batches of *D. odorifera.* Sativanone and 3′-*O*-methylviolanone were the dominant compounds in *D. odorifera*. The content of sativanone in all batches ranged from 5.8806 to 24.1200 mg/g (4.10-fold variation), and that of 3′-*O*-methylviolanone ranged from 0.6973 to 7.583 mg/g (10.87-fold variation). The content of daidzein in most samples was lower than the LOQ. For 3′-hydroxydaidein, genistein, and alpinetin, the contents were also relatively low (<0.2 mg/g). The trends observed in our results were similar to those in previous studies [15,16,17]. For example, Liu et al. [15] analyzed 10 major flavonoids in *D. odorifera* by HPLC-UV and found that sativanone (1.45–20.90 mg/g) was dominant. The average contents of other flavonoids (e.g., liquiritigenin, formononetin, and dalbergin) were higher than our results. In another study, seven flavonoids were analyzed in *D. odorifera* by Li et al. [17] and the obtained concentration ranges for the detected analytes (liquiritigenin, formononetin, isoliquiritigenin, and naringenin) were similar to those in the present study. Variation in the levels of flavonoids among the samples could be caused by differences in geographical conditions, the tree ages, plant origins, and storage conditions. The results suggest that UHPLC-MS/MS is a very powerful technique for quantitative analysis of multiple components of *D. odorifera* because it is rapid and sensitive. 

### 2.5. Fragmentation Pathways Analysis 

To date, 99 flavonoids have been isolated from *D. odorifera* [21]. These compounds have the same basic skeleton with different substituents. A total of 17 flavonoids, including six isoflavones (3′-hydroxydaidein, daidzein, genistein, tectorigenin, formononetin, and prunetin), five flavanones (liquiritigenin, eriodictyol, butin, naringenin, and pinocembrin), two chalcones (butein and isoliquiritigenin), two isoflavanones (sativanone and 3′-*O*-methylviolanone), one flavone (alpinetin), and one neoflavone (dalbergin) were quantified in the present study. Negative ion electrospray ionization (ESI) mode was found to be more sensitive than positive ion mode for detecting flavonoids. To further identify the compounds in *D. odorifera*, fragmentation pathways of six representative flavonoids (formononetin, pinocembrin, isoliquiritigenin, sativanone, alpinetin, and dalbergin) of *D. odorifera* were examined by UPLC-Q/TOF-MS/MS in negative ionization mode. 

**Figure 4:**
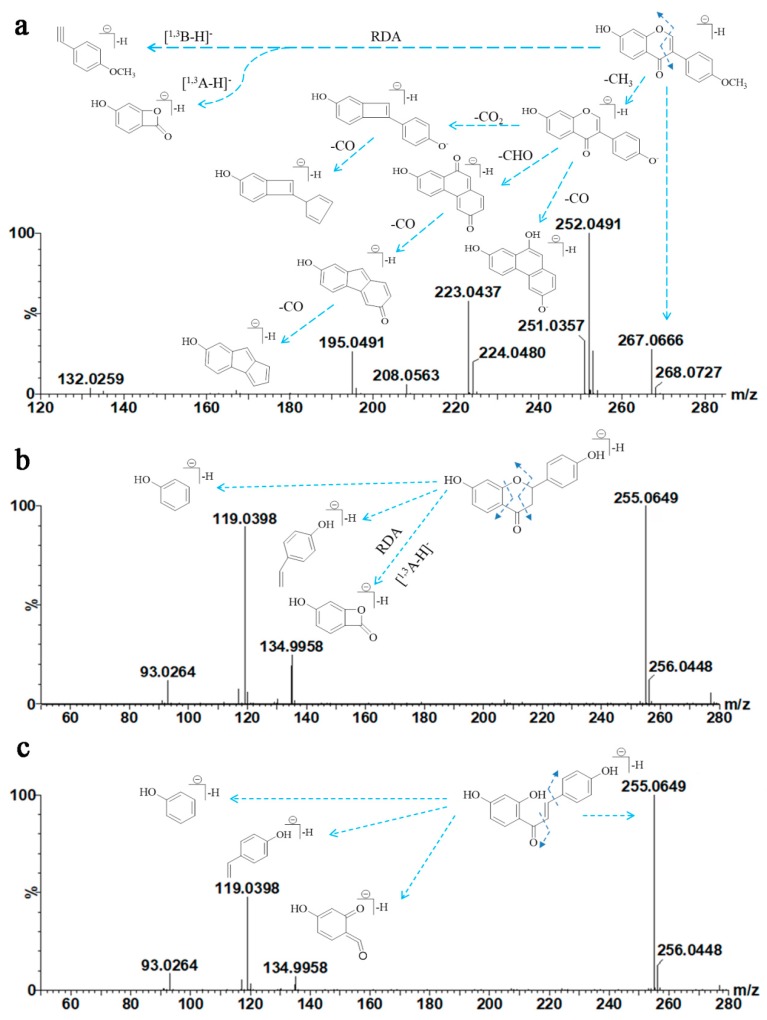
MS/MS spectra and the proposed fragmentation pathway of formononetin (**a**), liquiritigenin (**b**), and isoliquiritigenin (**c**).

Formononetin is a methoxylated isoflavone. The suggested fragmentation pathway of formononetin is shown in Figure 4a. The main and typical fragmentation ions of this compound result from successive or simultaneous losses of CH_3_, CHO, CO, and CO_2_, which are attributed to the 4′-OCH_3_ isoflavone type [24]. The base peak ion of formononetin at *m*/*z* 252.0491 [M − H-CH_3_]^−^ is formed by loss of a CH_3_ group. This result is consistent with a previous study that showed that loss of CH_3_ was characteristic of fragmentation in methoxylated flavonoids [25]. As the collision energy increased, abundant characteristic fragment ions were observed at *m*/*z* 223.0437 [M − H-CH_3_-CHO]^−^, *m*/*z* 224.0480 [M − H-CH_3_-CO]^−^, *m*/*z* 208.0563 [M − H-CH_3_-CO_2_]^−^, *m*/*z* 195.0491 [M − H-CH_3_-CHO-CO]^−^, *m*/*z* 180.0627 [M − H-CH_3_-CO_2_-CO]^−^, and *m*/*z* 167.0543 [M − H-CH_3_-CHO-2CO]^−^. It is worth mentioning that neutral loss of CO_2_ is common for isoflavones in MS/MS and the fragment ion at *m*/*z* 223.0437 differed from *m*/*z* 267.0666 by 44 Da, which is typically assigned as neutral loss of CO_2_. However, the TOF/MS revealed that the formula of *m*/*z* 223.0437 was C_14_H_7_O_3_, and this was formed by loss of C_2_H_4_O rather than CO_2_. Therefore, the typical loss of CO_2_ did not occur in this case. Instead, this fragment was produced via losses of CH_3_ and CHO at the 4′-position [26]. Fragment ions at *m*/*z* 132.0259 [^1,3^B − H]^−^ and 135.0125 [^1,3^A − H]^−^ were produced by retro-Diels–Alder (RDA) fragmentation in the C-ring of formononetin. 

Liquiritigenin gave a precursor ion [M − H]^−^ at *m*/*z* 255.0649 (Figure 4b). In the MS/MS spectrum, characteristic ions were observed at *m*/*z* 134.9958, 119.0398 and 93.0264, which were consistent with the typical [^1,3^A − H]^−^ and [^1,3^B − H]^−^ fragments. Isoliquiritigenin showed similar fragmentation behavior to liquiritigenin (Figure 4c).

**Figure 5:**
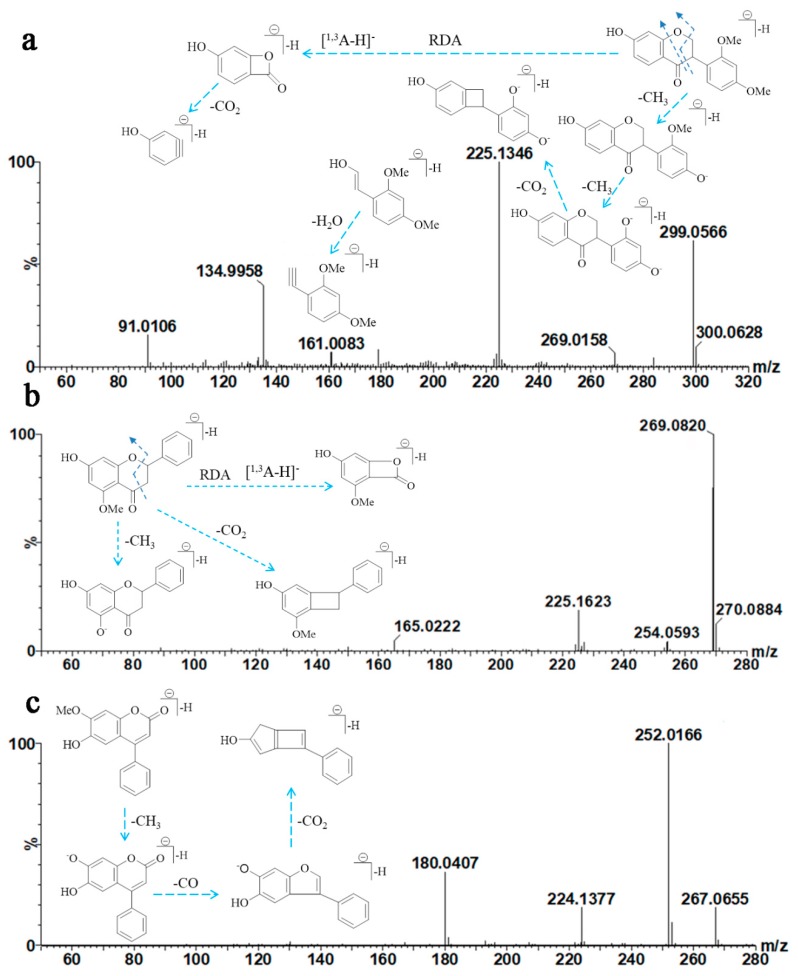
MS/MS spectra and the proposed fragmentation pathway of sativanone (**a**), alpinetin (**b**), and dalbergin (**c**).

The MS/MS spectra and fragmentation pathway of sativanone are shown in Figure 5a. Generally, losses of CH_3_ and CO_2_ were prominent. Loss of CH_3_ from the B-ring of sativanone yielded fragments at *m*/*z* 284.0719 [M − H-CH_3_] and *m*/*z* 269.0158 [M − H-CH_3_] produced from the precursor ion at 299.0566 ([M − H]^−^). Loss of CO_2_ from *m*/*z* 269.0158 yielded [M − H-2CH_3_-CO_2_]^−^ (*m*/*z* 225.1346). A fragment ion at *m*/*z* 134.9958 [^1,3^A − H]^−^ was generated after RDA cracking, and further loss of CO_2_ from *m*/*z* 134.9958 produced [^1,3^A − H-CO_2_]^−^ at *m*/*z* 91.0106. Additionally, fragmentation at the C-ring produced a ^0,3^B^−^ ion at *m*/*z* 179.0582 with low abundance. Further loss of one H_2_O produced an ion at *m*/*z* 161.0083. This fragmentation pathway was consistent with the previous report of Zhao et al. [27].

The fragmentation behavior for alpinetin is shown in Figure 5b. Alpinetin gave a [M − H]^−^ ion at *m*/*z* 269.0820 as the base peak. Two radical anions at *m*/*z* 254.0539 [M − H-CH_3_]^−^ and 225.1623 [M − H-CO_2_]^−^ formed by loss of CH_3_ and CO_2_ from the precursor anion. Additionally, a peak at *m*/*z* 165.0222 [^1,3^A − H]^−^ was observed after RDA fragmentation. 

Little research has been conducted on the fragmentation pathways of neoflavones [28]. The mass spectrum of dalbergin (Figure 5c) exhibited significant ions at *m*/*z* 267.0655, 252.0166, 224.1377, and 180.0407. The precursor ion lost one CH_3_ to give an ion at *m*/*z* 252.0166. Subsequent loss of CO from this ion generated a fragment ion *m*/*z* 224.1377. Further loss of CO_2_ from *m*/*z* 224.1377 yielded a fragment ion at *m*/*z* 180.0407. 

In negative ion ESI-MS/MS, all target analytes yielded prominent [M − H]^−^ ions. Some common features, such as loss of CH_3_, CO, and CO_2_, were observed in the MS/MS spectra, and were consistent with the literature. The [M − H-CH_3_]^−^ ion was a characteristic fragment of methoxylated flavonoids (formononetin, alpinetin, and dalbergin). In addition, [M − H-2CH_3_]^−^ fragments were observed for dimethoxylated flavonoids (sativanone). Therefore, loss of one or two CH_3_ could be adopted to identify single- or multi-methoxylated flavonoids in negative ion ESI-MS/MS. Loss of CO and CO_2_ from [M − H]^−^ ions was attributed to the structure of the C-ring. The [M − H]^−^ ions of formononetin, liquiritigenin, isoliquiritigenin, sativanone, and alpinetin underwent RDA fragmentation. However, RDA fragmentation was not observed for dalbergin, which may be related to its specific structural characteristics. 

## 3. Materials and Methods 

### 3.1. Solvents and Chemicals

HPLC-grade acetonitrile and formic acid were purchased from Thermo Fisher Scientific (Fair Lawn, NJ, USA). Analytical grade methanol was purchased from Beijing Chemical Works (Beijing, China). Deionized water was prepared using a Milli-Q system (Millipore, Milford, MA, USA). Reference standards of daidzein, dalbergin, 3′-hydroxydaidein, liquiritigenin, isoliquiritigenin, alpinetin, butein, naringenin, butin, prunetin, eriodictyol, and tectorigenin were purchased from Chengdu Chroma-Biotechnology Co., Ltd. (Chengdu, China). Rutin for use as an internal standard (IS), pinocembrin, formononetin, and genistein were obtained from Sichuan Victory Biological Technology Co., Ltd. (Chengdu, China). The purities of all standards were above 98.0%. We isolated sativanone and 3′-*O*-methylviolanone from the heartwood of *D. odorifera* T. Chen. The structures of these two compounds were unambiguously identified by NMR techniques, and their purities were determined to be above 96% by HPLC. 

Heartwood samples of *Dalbergia odorifera* T. Chen (*n* = 18) were collected from different areas in China. The samples were identified by Prof. Jianhe Wei (Institute of Medicinal Plant Development, Chinese Academy of Medical Science & Peking Union Medical College, Beijing, China), and voucher specimens (No. 469027-LD-020) were deposited in the Resource Center for Chinese Materia Media (Hainan Branch Institute of Medicinal Plant Development, Chinese Academy of Medical Sciences & Peking Union Medical College, China).

### 3.2. UHPLC-QqQ-MS/MS

Analyses were performed on a UHPLC system (Acquity H-Class, Waters Corp., Milford, MA, USA) with a binary solvent manager, a column manager, and a sample manager. The sample was separated on a Waters Acquity HSS T3 column (100 mm × 2.1 mm, 1.8 μm; Waters Corp.) and the column temperature was set at 40 °C. The mobile phase consisted of acetonitrile (A) and water containing 0.05% formic acid (B) with a flow rate of 0.25 mL/min. The following gradient program was used: 5–30% A from 0–2 min, 30–33% A from 2–5 min, 33–53% A from 5–13 min, held at 53% A for 3 min, 53–95% A from 16–18 min, held at 95% A for 2 min, and 95–5% A from 20–22 min. For UHPLC-MS/MS analysis, a Waters QqQ-MS (Xevo TQ-D, Waters Corp.) was connected to the Waters UHPLC instrument via an electrospray ionization (ESI) source. Analytes were quantified by MRM in negative ionization mode with argon as the collision gas. All MS parameters were optimized in the combined mode. The following ESI ion source parameters were used: capillary voltage, 2.5 kV; source temperature, 150 °C; desolvation temperature, 400 °C; cone gas (nitrogen) flow rate, 50 L/h; and desolvation gas (nitrogen) flow rate, 600 L/h. The UHPLC-MS/MS parameters, including the precursor-to-product ion transitions, cone voltage, and collision energy, are listed in Table 1.

### 3.3. UHPLC-Q/TOF-MS/MS

Flavonoid fragmentation was performed on a quadrupole time-of-flight mass spectrometer (Xevo G2-XS, Waters Corp.) equipped with an ESI source and coupled to the UPLC system. The above UHPLC conditions were used for UHPLC-Q/TOF-MS/MS. Detection was implemented in the MS^E^ centroid mode over a mass range of 500–1000 Da with a scan rate of 10 Da/s. The analyzer sensitivity mode was used. Leukine enkephalin was infused using LockSpray via a reference probe for in-run mass corrections. The ESI ion source parameters were as follows: capillary voltage, 2.5 kV; desolvation gas (nitrogen) flow rate, 600 L/h, desolvation temperature, 400 °C; cone gas flow rate, 50 L/h; and source temperature, 150 °C. The collision energy was ramped in a high energy function from 20 to 60 eV using argon as the collision gas. MassLynx software (Waters Corp.) was used for post-acquisition analysis.

### 3.4. Sample Preparation

The materials were pulverized and dried to a constant mass before use. A 0.20 g sample was extracted with 25 mL of 70% methanol (*v*/*v*) in an ultrasonic water bath for 45 min and then filtered. An aliquot (1 mL) of the filtrate was transferred into a 15-mL screw cap plastic tube containing 9 mL of 70% aqueous methanol and shaken immediately for 1 min using a vortex mixer. Then, 0.5 mL of IS (1.0 μg/mL) was added to 0.5 mL of the solution and the mixture was vortexed for 30 s. The obtained solution was filtered through a 0.22-μm micropore membrane before use. A 5 μL sample was injected into the UHPLC instrument for analysis. 

### 3.5. Standard Solution Preparation

Standard stock solutions of 17 reference standards with a concentration range of 24.4 to 113.04 μg/mL were separately prepared by dissolution in methanol. An initial stock solution was prepared as a mixture of the above stock solutions. A series of working solutions of the analytes were obtained by diluting the mixed stock solution with methanol to the appropriate concentration. Then, 0.5 mL of IS (1.0 μg/mL) was added to 0.5 mL of the working solutions and the mixture was vortexed for 30 s. All of the solutions were stored at 4 °C and filtered through a 0.22-μm nylon membrane before injection into the UHPLC system. 

## 4. Conclusions

In the present study, a sensitive and rapid UHPLC-ESI-MS/MS method was established for the simultaneous quantitative analysis of 17 flavonoids in the heartwood of *D. odorifera* and successfully applied to 18 samples. Satisfactory validation parameters were obtained, including specificity, linearity, precision, accuracy, and stability, and the extraction method was optimized. Taking the contents of the target compounds into consideration, the quantification results indicated that the quality of *D. odorifera* varied. The MS fragmentation pathways of flavonoids discussed here could facilitate rapid screening and structural characterization of these compounds by LC-MS/MS. Our results suggest that UHPLC-MS/MS could be a useful tool for quality assessment of *D. odorifera* using flavonoids as markers.

## Figures and Tables

**Table 1:** MS/MS parameters for 17 target compounds.

No.	Compounds	Ion Mode	RT (min)	Precursor Ion	Cone Voltage (V)	Product ion 1 ^Q^	Collision Energy (eV)	Product Ion 2 ^I^	Collision Energy (eV)
1	3′-Hydroxydaidein	ESI^-^	4.97	269	52	213	28	241	24
2	Butein	ESI^-^	5.12	271	35	135	20	253	17
3	Daidzein	ESI^-^	5.95	253	51	208	31	224	26
4	Liquiritigenin	ESI^-^	6.23	255	40	135	15	119	22
5	Eriodictyol	ESI^-^	6.24	287	38	151	17	135	24
6	Butin	ESI^-^	7.59	271	39	135	31	91	37
7	Naringenin	ESI^-^	7.94	271	40	151	20	119	24
8	Genistein	ESI^-^	8.04	269.	48	133	32	181	28
9	Tectorigenin	ESI^-^	8.30	2989	40	284	19	240	22
10	Alpinetin	ESI^-^	8.65	269	44	165	20	227	21
11	Isoliquiritigenin	ESI^-^	9.62	255	35	135	15	119	23
12	Formononetin	ESI^-^	10.08	267	45	252	22	223	25
13	Dalbergin	ESI^-^	10.09	267	38	180	27	252	18
14	3′-*O*-methylviolanone	ESI^-^	10.62	329	46	135	38	299	36
15	Sativanone	ESI^-^	11.10	299	46	135	37	269	32
16	Pinocembrin	ESI^-^	12.47	255	42	107	25	171	25
17	Prunetin	ESI^-^	12.97	283	45	268	21	239	26
18	Rutin (IS)	ESI^-^	4.05	609	62	300	52	271	50

^Q^: transitions for quantification; ^I^: transitions for identification.

**Table 2:** Curves, test range, limit of detection (LOD), limit of quantification (LOQ), precision, and repeatability for the seventeen analytes.

No.	Compounds	Calibration Curves	*r* ^2^	Linear Range (ng/mL)	LOQ (ng/mL)	LOD (ng/mL)	Precision (RSD, %)		Repeatability
							Intra-Day	Inter-Day	(RSD, %, *n* = 6)
1	3′-Hydroxydaidein	*Y* = 0.934*X* − 0.0436	0.9991	5.40–1350	5.400	1.600	2.43	3.23	3.45
2	Butein	*Y* = 0.1675*X* − 0.198	0.9993	1.41–2820	1.410	0.470	2.49	4.85	4.53
3	Daidzein	*Y* = 0.9697*X* − 0.061	0.9989	3.02–1510	3.020	1.000	1.74	3.01	2.98
4	Liquiritigenin	*Y* = 0.1813*X* + 0.0075	0.9999	1.61–3220	1.610	0.500	1.25	2.26	3.18
5	Eriodictyol	*Y* = 0.1802*X* − 0.0166	0.9997	1.36–1360	1.360	0.453	2.38	2.45	2.06
6	Butin	*Y* = 0.1163*X* − 0.0568	0.9986	1.51–3020	1.510	0.458	1.85	3.54	2.77
7	Naringenin	*Y* = 0.2291*X* − 0.0722	0.9989	2.72–1360	2.720	0.906	2.07	4.61	4.06
8	Genistein	*Y* = 0.8139*X* − 0.2152	0.9988	3.82–1910	3.820	1.528	0.76	3.33	2.54
9	Tectorigenin	*Y* = 0.203*X* − 0.2161	0.9987	2.44–1220	2.440	0.813	1.96	2.40	1.95
10	Alpinetin	*Y* = 0.5127*X* − 0.0544	0.9996	5.36–1340	5.360	1.790	2.85	4.94	4.76
11	Isoliquiritigenin	*Y* = 0.1308*X* + 0.0284	0.9996	1.416–1770	1.416	0.480	0.45	3.02	3.67
12	Formononetin	*Y* = 0.0516*X* − 0.0608	0.9993	0.516–1290	0.516	0.172	1.78	1.90	3.32
13	Dalbergin	*Y* = 0.2867*X* − 0.0665	0.9991	0.256–1280	0.256	0.085	3.51	4.85	4.61
14	3′-*O*-methylviolanone	*Y* = 0.6244*X* + 0.0119	0.9989	9.90–2970	9.900	3.300	2.08	4.49	2.87
15	Sativanone	*Y* = 0.675*X* − 0.1047	0.9991	18.84–5652	18.840	6.080	1.24	1.26	4.73
16	Pinocembrin	*Y* = 0.3485*X* − 0.0569	0.9992	2.66–1330	2.660	0.870	1.04	1.94	3.82
17	Prunetin	*Y* = 0.0489*X* − 0.0657	0.9989	1.12–2240	1.120	0.374	1.91	3.34	3.61

**Table 3:** Contents of 17 analytes in 18 batches of samples (mg/g).

	1	2	3	4	5	6	7	8	9	10	11	12	13	14	15	16	17
S1	0.1004	0.0769	0.0546	0.7228	<LOQ	1.7084	0.3902	0.0396	0.9048	ND	0.2994	1.1047	0.0255	0.6973	5.8806	0.1901	0.1207
S2	0.1052	0.3714	0.0422	0.8499	0.0424	0.2862	0.3362	0.0302	0.7439	ND	0.4019	1.0490	0.0452	1.4732	6.5233	0.2467	0.1119
S3	0.0232	0.4628	<LOQ	0.6125	0.3140	0.2346	0.6570	0.0379	0.2066	0.0436	0.2881	0.2712	0.1724	1.8881	8.7726	0.8209	0.1794
S4	0.1340	0.0865	0.1102	1.0619	<LOQ	0.0400	0.3603	0.0828	0.8650	0.0860	0.5860	1.2523	0.0171	0.8051	9.5705	0.2324	0.1228
S5	<LOQ	0.4860	<LOQ	0.1908	0.5322	0.2570	0.8737	0.0234	0.1184	0.0141	0.1192	0.0667	0.0302	7.5830	18.2890	0.2874	0.0806
S6	0.0281	0.9359	<LOQ	0.3571	0.5365	0.5594	0.5046	0.0226	0.1325	0.0241	0.1852	0.3650	0.0164	3.8599	24.1200	0.3118	0.1195
S7	<LOQ	0.4820	<LOQ	0.2767	0.0364	0.2680	0.1307	0.0068	0.0751	0.0310	0.1744	0.1930	0.0701	1.0216	7.2718	0.0500	0.0147
S8	0.0366	0.7531	<LOQ	0.5434	0.0658	0.5064	0.2038	0.0251	0.1711	0.0665	0.3136	0.3630	0.1084	1.4806	7.3738	0.1676	0.0381
S9	0.0888	0.9047	0.0442	0.8766	0.5470	0.5149	1.1659	0.0688	0.2205	0.0258	0.4645	0.7756	0.4961	2.8056	12.6911	1.3608	0.3284
S10	0.0423	0.8399	<LOQ	0.5717	0.4619	0.5250	0.7914	0.0393	0.3628	0.0239	0.3086	0.6162	0.3954	3.5777	14.6963	1.1722	0.2915
S11	0.0961	0.9512	0.0380	1.0640	0.0907	0.6402	0.2374	0.0079	0.2600	0.0130	0.5423	0.7814	0.4026	1.6566	6.7257	0.4049	0.0701
S12	0.0375	0.6593	<LOQ	1.0338	0.7855	0.4597	1.5180	0.0820	0.4318	0.0226	0.6463	0.4233	0.0024	3.3384	15.1491	1.2503	0.4409
S13	0.0431	1.0001	<LOQ	0.8340	1.1740	0.6573	2.4237	0.1641	0.2210	0.0375	0.4746	0.6412	0.1302	4.5177	23.7313	2.1126	0.5725
S14	0.0625	1.9857	<LOQ	1.8790	0.0830	1.6506	0.1734	ND	0.2656	0.0111	1.3703	1.1935	0.4145	1.0923	8.3085	0.1969	0.0464
S15	0.0621	0.9035	<LOQ	0.5811	0.2317	0.4897	0.7012	0.0414	0.2702	0.0211	0.3043	0.6900	0.1995	2.5788	15.1260	0.5150	0.2174
S16	0.0536	0.5246	0.0399	0.4060	0.0321	0.2395	0.1328	0.0161	0.1031	0.0131	0.2361	0.3781	0.1192	0.8817	6.6720	0.1057	0.0315
S17	<LOQ	0.2551	0.0544	0.6073	<LOQ	0.1680	0.3325	0.0300	0.1935	0.0161	0.3011	0.4070	0.0034	1.5828	21.6877	0.3601	0.0606
S18	ND	0.1246	ND	0.1123	0.0243	0.0460	0.1787	0.0053	0.0580	0.0869	0.0719	0.0731	0.0252	1.1494	8.3910	0.1419	0.0325

## Supplementary Materials

The following are available online, Table S1: Recoveries of 17 target compounds.
