# Supplementary material for: Analysis of Flavonoids in Dalbergia odorifera by Ultra-Performance Liquid Chromatography with Tandem Mass Spectrometry
Source: Molecules. 2020 Jan 17;25(2):389. doi: 10.3390/molecules25020389 (PMC7024225; doi:10.3390/molecules25020389)
Supplement: Supplementary file 1 [file molecules-25-00389-s001.pdf]

Analysis of flavonoids in *Dalbergia odorifera* by ultra-performance liquid chromatography with tandem mass spectrometry

Xiangsheng Zhao[1], Shihui Zhang[1], Dan Liu[3], Meihua Yang[1,2], Jianhe Wei[1,2*]

[1] *Hainan Provincial Key Laboratory of Resources Conservation and Development of Southern Medicine, Hainan Branch of the Institute of Medicinal Plant Development, Chinese Academy of Medical Sciences & Peking Union Medical College, Haikou 570311, China;*
[2] *Institute of Medicinal Plant Development, Chinese Academy of Medical Sciences & Peking Union Medical College, Beijing 100193,China;*
[3] *School of Life Science and Engineering, Southwest University of Science and Technology, Mianyang, 621010, China;*

*Corresponding author
Jianhe Wei, Institute of Medicinal Plant Development, Chinese Academy of Medical Sciences & Peking Union Medical College, No. 151 Malianwa North Road, Haidian District, Beijing 100193 P. R. China; Tel.: +8610 57833358; Fax: +8610 57833359; E-mail: wjianh@263.net.

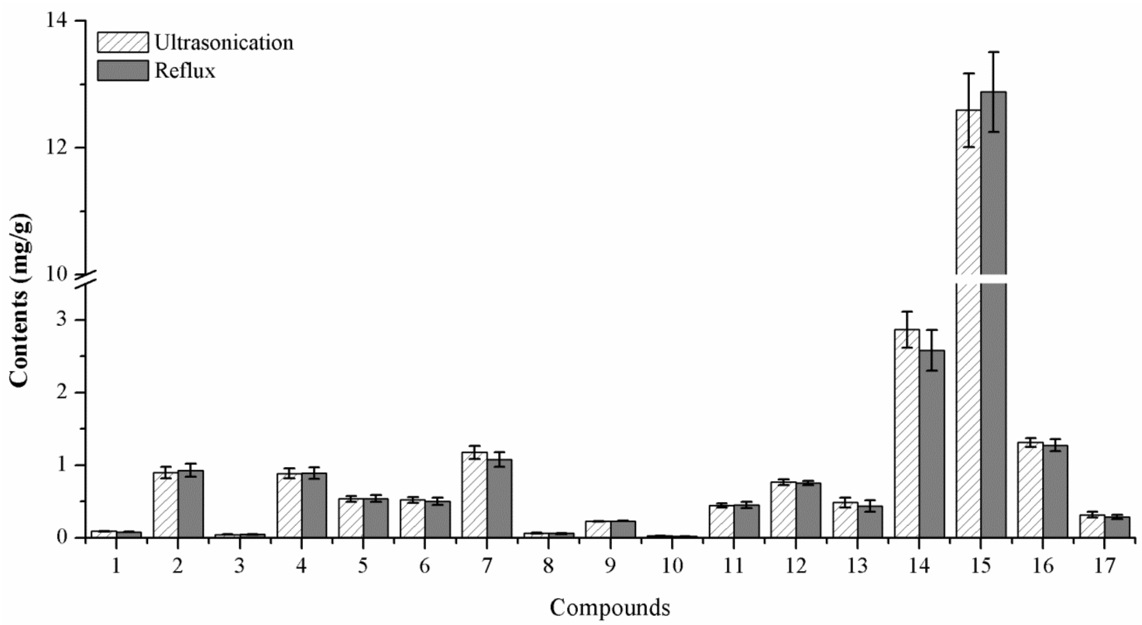

Figure S1. Effects of extraction method on the extraction efficiency of the target analytes in S9 samples.

Table S1. Recoveries of 17 target compounds

NO.	Analytes	Added (μg)	Detected (μg)	Recovery (%)	RSD (%)
1	3'-Hydroxydaidein	11.00	10.66	96.94	3.17
		9.00	8.68	96.46	2.69
		7.00	6.84	97.69	3.89
2	Butein	110.00	106.88	97.16	2.68
		90.00	88.32	98.13	3.05
		75.00	73.31	97.74	3.73
3	Daidzein	5.50	5.23	95.18	5.01
		4.50	4.43	98.37	4.43
		3.50	3.42	97.66	3.81
4	Liquiritigenin	110.00	109.21	99.28	3.52
		90.00	88.60	98.44	2.88
		70.00	68.75	98.21	3.65
5	Eriodictyol	65.00	62.47	96.11	3.15
		55.00	52.54	95.52	4.72
		45.00	43.04	95.65	3.80
6	Butin	65.00	63.08	97.04	2.74
		50.00	47.09	94.18	3.98
		45.00	43.37	96.38	2.63
7	Naringenin	140.00	136.11	97.22	3.19
		120.00	114.44	95.37	1.93
		95.00	92.06	96.91	2.47
8	Genistein	8.50	8.39	98.65	1.86
		7.00	6.87	98.15	2.70
		5.50	5.34	97.10	1.99
9	Tectorigenin	25.00	23.90	95.59	3.53
		20.00	19.22	96.09	4.12
		16.00	15.61	97.58	3.03
10	Alpinetin	3.00	2.83	94.32	2.03
		2.50	2.38	95.32	3.34
		2.00	1.89	94.65	2.95
11	Isoliquiritigenin	55.00	54.91	99.84	2.87
		45.00	44.81	99.57	4.61
		40.00	39.41	98.53	3.98
12	Formononetin	95.00	96.87	101.97	3.61
		80.00	79.06	98.82	4.58
		60.00	59.59	99.31	3.86
13	Dalbergin	60.00	59.35	98.91	3.66
		50.00	49.63	99.25	2.48
		40.00	39.23	98.07	3.20
14	3'-*O*-methylviolanone	350.00	344.23	98.35	4.17
		300.00	299.19	99.73	3.49
		225.00	217.73	96.77	3.25

15	Sativanone	1500.00	1449.15	96.61	2.96
		1250.00	1220.38	97.63	3.15
		1000.00	954.80	95.48	1.88
16	Pinocembrin	160.00	153.98	96.24	4.75
		140.00	133.80	95.57	4.23
		110.00	108.10	98.27	5.22
17	Prunetin	40.00	39.24	98.10	3.81
		32.00	31.94	99.81	4.52
		25.00	23.79	95.15	3.65
